# Potential of long non‐coding RNA KCNQ1OT1 as a biomarker reflecting systemic inflammation, multiple organ dysfunction, and mortality risk in sepsis patients

**DOI:** 10.1002/jcla.24047

**Published:** 2021-11-10

**Authors:** Wei Jiao, Xin Zhou, Jian Wu, Xuesong Zhang, Jun Ding

**Affiliations:** ^1^ Department of Nursing No. 904th Hospital of The Joint Logistics Support Force of the PLA Wuxi China; ^2^ Department of Clinical Laboratory No. 904th Hospital of The Joint Logistics Support Force of the PLA Wuxi China; ^3^ Department of Information No. 904th Hospital of The Joint Logistics Support Force of the PLA Wuxi China; ^4^ Department of Anesthesiology Shanghai Public Health Clinical Center Shanghai China; ^5^ Department of Urology No. 904th Hospital of The Joint Logistics Support Force of the PLA Wuxi China

**Keywords:** inflammation, Lnc‐KCNQ1OT1, mortality risk, multiple organ dysfunction, sepsis

## Abstract

**Background:**

Long non‐coding RNA potassium voltage‐gated channel subfamily Q member 1 opposite strand 1 (lnc‐KCNQ1OT1) represses inflammation and multiple organ dysfunction, whereas its clinical value in sepsis is unclear. Thus, this study aimed to explore this issue.

**Methods:**

Lnc‐KCNQ1OT1 from peripheral blood mononuclear cells were detected by RT‐qPCR in 116 sepsis patients and 60 healthy controls (HCs). Moreover, sepsis patients were followed‐up until death or up to 28 days.

**Results:**

Lnc‐KCNQ1OT1 decreased in patients with sepsis than in HCs (*p* < 0.001). In sepsis patients, lnc‐KCNQ1OT1 was negatively correlated with sequential organ failure assessment (SOFA) scores (*r* = −0.344, *p* < 0.001) and several SOFA subscale scores (including respiratory system, coagulation, liver, and renal systems) (all *r* < 0, *p* < 0.05). Furthermore, lnc‐KCNQ1OT1 was negatively correlated with CRP (*r* = −0.386, *p *< 0.001), TNF‐α (*r* = −0.332, *p* < 0.001), IL‐1β (*r* = −0.319, *p* < 0.001), and IL‐6 (*r* = −0.255, *p* = 0.006). Additionally, lnc‐KCNQ1OT1 levels were lower in sepsis deaths than in sepsis survivors (*p* < 0.001), and the receiver operating characteristic curve showed that lnc‐KCNQ1OT1 had an acceptable ability to predict 28‐day mortality (area under the curve: 0.780, 95% confidence interval: 0.678–0.882). Meanwhile, its ability to predict 28‐day mortality risk was higher than that of CRP, TNF‐α, IL‐1β, and IL‐6, but slightly lower than the SOFA score and acute physiology and chronic health evaluation II score.

**Conclusion:**

Lnc‐KCNQ1OT1 serves as a potential biomarker for monitoring disease severity and prognosis in patients with sepsis.

## Graphical abstract

Lnc‐KCNQ1OT1 negatively correlated with multiple organ dysfunction and inflammatory indexes in sepsis patients. Furthermore, lnc‐KCNQ1OT1 decreased in sepsis deaths compared to sepsis survivors, whose expression could discriminate sepsis deaths from sepsis survivors.

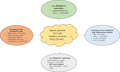

## 1 INTRODUCTION

Sepsis is a life‐threatening disease induced by dysfunctional responses to infection.^1^ Meanwhile, inflammatory storm and multiple organ dysfunction (such as lung, kidney, liver, cardiac, and nervous system) are hallmarks of sepsis.^2^ Furthermore, sepsis affects approximately 18 million people worldwide and continues to be the major contributor to infection‐induced death globally (especially in critically ill patients), which results in huge economic and disease burdens.^2^, ^3^, ^4^ Considering that the prognosis of sepsis patients is still poor and that mortality continues to climb, the exploration of novel biomarkers to improve the management of sepsis is important.^2^, ^3^, ^5^

Long non‐coding RNA potassium voltage‐gated channel subfamily Q member 1 (KCNQ1) opposite strand 1 (lnc‐KCNQ1OT1) has been shown to suppress inflammation and multiple organ dysfunction.^6^, ^7^, ^8^, ^9^ For instance, lnc‐KCNQ1OT1 is able to inhibit inflammation through nuclear factor kappa B inhibitor alpha (IκBα) and regulating microRNA (miR)‐506‐3p,^6^, ^8^, ^9^ while lnc‐KCNQ1OT1 has the capacity to attenuate multiple organ dysfunction (such as cardiomyopathy, liver injury, and sepsis‐induced cardiac injury) via several approaches, including regulation of miR‐214‐3p, caspase‐1, miR‐122‐5p and carboxylesterase 2, as well as miR‐192‐5p and the X‐linked inhibitor of apoptosis protein (XIAP) axis.^7^, ^10^, ^11^ Based on this information, we speculated that lnc‐KCNQ1OT1 levels might be correlated with inflammation and multiple organ dysfunction in sepsis, while the relevant data are obscured.

Therefore, the present study aimed to explore potential correlations involving lnc‐KCNQ1OT1 and inflammation, multiple organ dysfunction, and mortality risk among sepsis patients.

## 2 METHODS

### 2.1 Subjects

A total of 116 sepsis patients treated in our hospital from February 2018 to June 2020 were consecutively enrolled in this prospective study. The enrollment criteria were as follows: (i) diagnosis of sepsis according to the Third International Consensus Definitions for Sepsis^12^; (ii) aged >18 years; and (iii) were admitted to our department within 24 h after the onset of symptoms. Patients were ineligible for inclusion if they had experienced the following conditions: (i) complications involving carcinomas or blood malignancies, (ii) concomitant autoimmune diseases, (iii) used immunosuppressants before enrollment, (iv) received chemotherapy within 3 months, (v) pregnancy and lactating women, and (vi) poor study compliance. In addition, 60 healthy subjects who underwent physical examination in our hospital from January 2020 to June 2020 were recruited as healthy controls (HCs). The recruitment criteria for HCs were as follows: (i) age‐ and sex‐matched to sepsis patients, (ii) no history of sepsis or severe infection, and (iii) had normal biochemical index levels. HCs were also excluded from the study if they met the exclusion criteria for sepsis patients. This study was approved by the Institutional Review Board of No. 904th Hospital of The Joint Logistics Support Force of the PLA, and all participants or their relatives signed informed consent forms.

### 2.2 Data documentation

Demographics, comorbidities, disease characteristics, and biochemical indices were recorded after clinical and laboratory examinations. Acute Physiology and Chronic Health Evaluation II (APACHE II) score and Sequential Organ Failure Assessment (SOFA) scores were assessed within 24 h of hospitalization to evaluate the disease status of patients. All sepsis patients were closely followed‐up until death or for up to 28 days, and deaths within 28 days were recorded.

### 2.3 Peripheral blood (PB) collection

PB was sampled from sepsis patients immediately upon admission and from HCs after enrollment. Peripheral blood mononuclear cells (PBMCs) and serum were separated from the PB samples by density gradient centrifugation.

### 2.4 Lnc‐KCNQ1OT1 determination

Quantitative reverse‐transcription polymerase chain reaction (RT‐qPCR) assay was carried out to analyze the expression of lnc‐KCNQ1OT1 in PBMCs. In brief, total RNA was extracted by QIAamp RNA Blood Mini Kit (Qiagen, Duesseldorf, Nordrhein‐Westfalen, Germany) and reverse‐transcribed using iScript™ cDNA Synthesis Kit with oligo d(T) and random hexamer primers (Bio‐Rad, Hercules, California, USA). qPCR was performed using SYBR^®^ Green Real‐time PCR Master Mix (Toyobo, Osaka, Kansai, Japan). Relative expression was calculated by the 2^−ΔΔCt^ method, and *GAPDH* was used as an internal reference. Primers used for PCR amplification were designed according to a previous study.^13^

### 2.5 Inflammatory cytokine determination

Tumor necrosis factor alpha (TNF‐α), interleukin‐1β (IL‐1β), and interleukin‐6 (IL‐6) in sera of patients with sepsis were determined by enzyme‐linked immunosorbent assay (ELISA). All ELISA kits were purchased from Bio‐Techne China Co., Ltd. (catalog number: DTA00D, DLB50, D6050; R&D Systems, Shanghai, China). All ELISA procedures were performed in strict accordance with the experimental protocol recommended by the manufacturer.

### 2.6 Statistical analysis

SPSS (v.21.0; IBM Corp., Armonk, New York, USA) and GraphPad Prism v.6.01 software (GraphPad Software Inc., San Diego, CA, USA) were employed to perform statistical analysis and graph plotting, respectively. The Mann‐Whitney *U* test was used to compare lnc‐KCNQ1OT1 expression between the two groups. Correlations between lnc‐KCNQ1OT1 expression and clinical data were analyzed using Spearman's rank correlation test. The performance of variables in evaluating mortality risk was estimated using receiver operating characteristic (ROC) curve analysis. Statistical significance was determined for *p* values <0.05 in the corresponding analyses.

## 3 RESULTS

### 3.1 Clinical features

Among 116 sepsis patients, the mean age was 58.4 ± 13.4 years, and 77 (66.4%) were male. In addition, the mean APACHE II score was 12.1 ± 6.5 and the mean SOFA score was 5.5 ± 2.7. In terms of biochemical indices, the median values of C‐reactive protein (CRP), TNF‐α, IL‐1β, and IL‐6 were 117.0 (62.4–171.0) mg/L, 192.5 (120.8–288.1) pg/ml, 11.3 (6.0–17.9) pg/ml, and 98.8 (50.2–157.9) pg/ml, respectively. More detailed clinical features of patients with sepsis are presented in Table 1.

**TABLE 1:** Sepsis patients’ characteristics.

Items	Sepsis patients (*N* = 116)
Demographic characteristics	
Age (years), mean±SD	58.4 ± 13.4
Male, No. (%)	77 (66.4)
BMI (kg/m^2^), mean±SD	23.3 ± 3.6
History of smoking, No. (%)	46 (39.7)
History of drinking, No. (%)	43 (37.1)
Comorbidities, No. (%)	
Hypertension	41 (35.3)
CCVD	25 (21.6)
Hyperlipidemia	19 (16.4)
Diabetes	19 (16.4)
CKD	9 (7.8)
Disease characteristics	
Primary infection site, No. (%)	
Abdominal infection	48 (41.4)
Respiratory infection	30 (25.9)
Skin and soft tissue infection	24 (20.7)
Other infections	14 (12.1)
Primary organism, No. (%)	
G‐	57 (49.1)
G+	36 (31.0)
Fungus	12 (10.3)
Others	22 (19.0)
Negative culture	18 (15.5)
APACHE II score, mean±SD	12.1 ± 6.5
SOFA score, mean±SD	5.5 ± 2.7
Respiratory system	1.4 ± 0.7
Coagulation	1.1 ± 0.6
Liver	0.7 ± 0.7
Cardiovascular system	0.7 ± 0.7
Nervous system	0.6 ± 0.5
Renal system	1.1 ± 0.7
Biochemical indexes	
CRP (mg/L), median (IQR)	117.0 (62.4–171.0)
TNF‐α (pg/ml), median (IQR)	192.5 (120.8–288.1)
IL−1β (pg/ml), median (IQR)	11.3 (6.0–17.9)
IL−6 (pg/ml), median (IQR)	98.8 (50.2–157.9)

Abbreviations

APACHE II, Acute Physiology and Chronic Health Evaluation II; BMI, body mass index; CCVD, cardiovascular and cerebrovascular diseases; CKD, chronic kidney disease; CRP, C‐reactive protein; SD, standard deviation; SOFA, Sequential Organ Failure Assessment; IL‐1β, interleukin‐1beta; IL‐6, interleukin 6; IQR, interquartile range; TNF‐α, tumor necrosis factor alpha.

### 3.2 Comparison of lnc‐KCNQ1OT1 between sepsis patients and HCs

**FIGURE 1:**
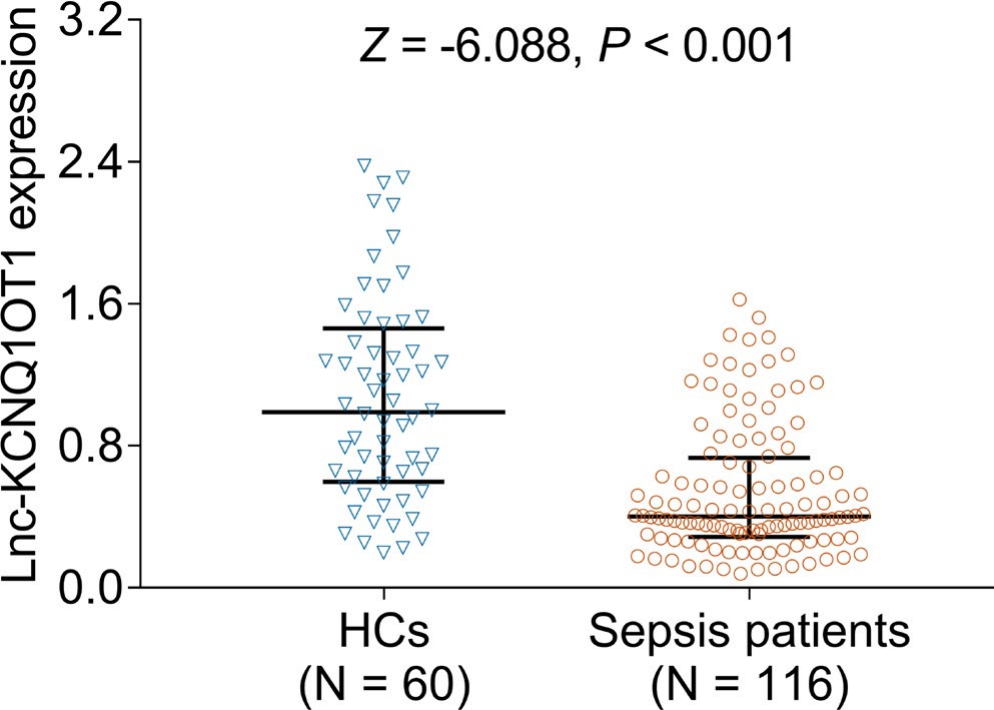
lnc‐KCNQ1OT1 in sepsis patients and HCs. lnc‐KCNQ1OT1, long non‐coding RNA potassium voltage‐gated channel subfamily Q member 1 (KCNQ1) opposite strand 1; HC, healthy control

To determine the expression of lnc‐KCNQ1OT1 in sepsis patients and HCs, RT‐qPCR was performed in the present study. We found that lnc‐KCNQ1OT1 levels were lower in sepsis patients (median [interquartile range, IQR]: 0.402 [0.288–0.732]) than in HCs (median [IQR]: 0.990 [0.596–1.462]) (*p* < 0.001) (Figure 1).

### 3.3 Correlation of lnc‐KCNQ1OT1 abundance with SOFA score and subscales

**FIGURE 2:**
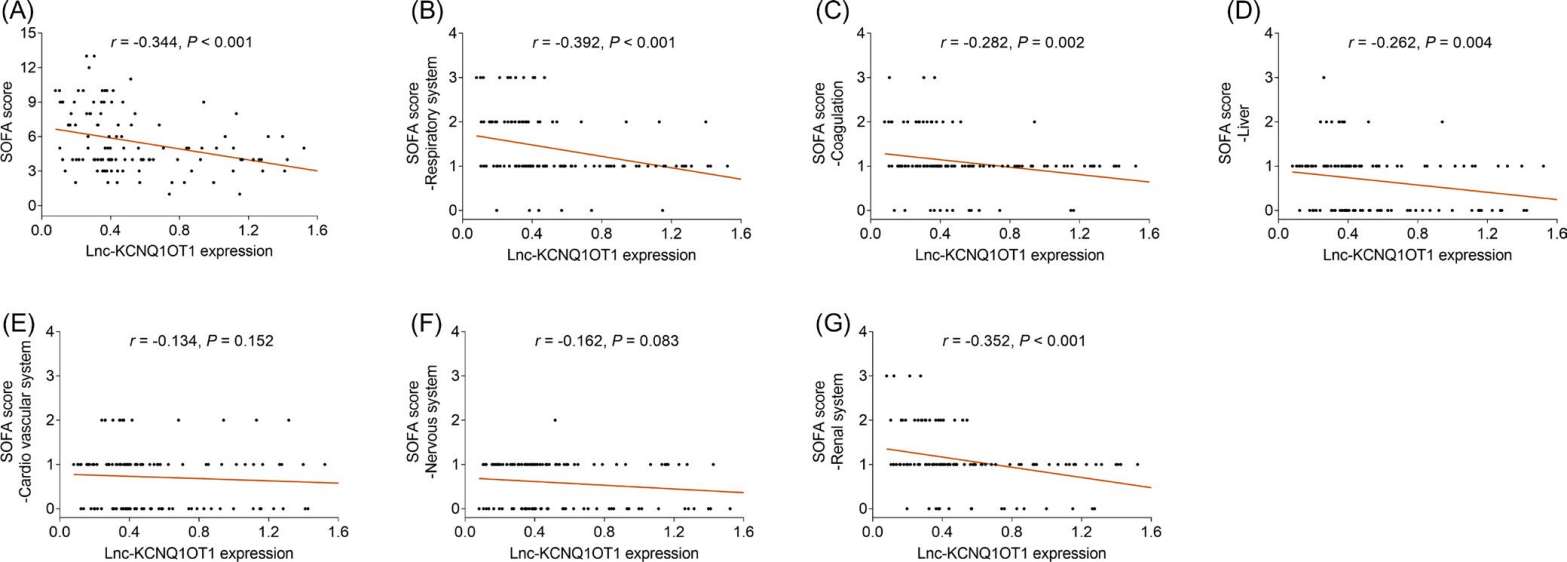
Association of lnc‐KCNQ1OT1 levels with SOFA scores. Association of lnc‐KCNQ1OT1 expression with SOFA scores (A), SOFA score‐respiratory system (B), SOFA score‐coagulation (C), SOFA score‐liver (D), SOFA score‐ cardiovascular system (E), SOFA score‐nervous system (F), and SOFA score‐renal system (G). SOFA, sequential organ failure assessment; lnc‐KCNQ1OT1, long non‐coding RNA potassium voltage‐gated channel subfamily Q member 1 (KCNQ1) opposite strand 1

Lnc‐KCNQ1OT1 was negatively correlated with SOFA scores (*r* = −0.344, *p* < 0.001) (Figure 2A). Furthermore, regarding the correlation between lnc‐KCNQ1OT1 and SOFA subscale scores, a negative correlation was found between lnc‐KCNQ1OT1 and SOFA score‐respiratory system (*r* = −0.392, *p* < 0.001), SOFA score‐coagulation (*r *= −0.282, *p* = 0.002), SOFA score‐liver (*r* = −0.262, *p* = 0.004), and SOFA score‐renal system (*r* = −0.352, *p* < 0.001) (Figure 2B–D,G); however, no statistically significant correlation was found for lnc‐KCNQ1OT1 with SOFA score‐cardio vascular system or SOFA score‐nervous system (both *p* > 0.05) (Figure 2E,F).

### 3.4 Correlation of lnc‐KCNQ1OT1 with inflammatory indices and other clinical features

**FIGURE 3:**
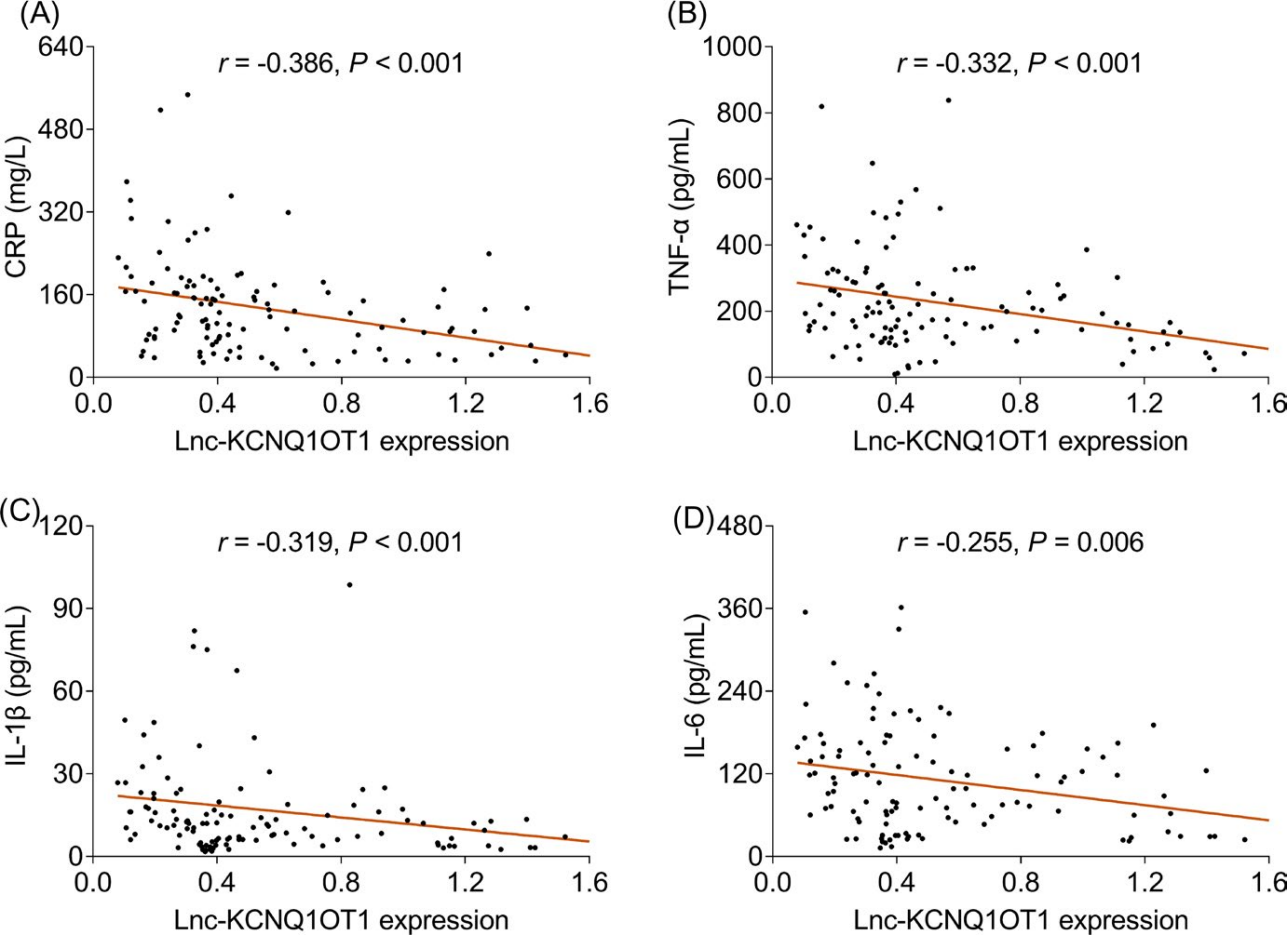
Association of lnc‐KCNQ1OT1 with inflammatory indices. Association of lnc‐KCNQ1OT1 expression with CRP (**A**), TNF‐α (**B**), IL‐1β (**C**), and IL‐6 (**D**). CRP, C‐reactive protein; TNF‐α, tumor necrosis factor‐alpha; IL‐1β, interleukin‐1β; IL‐6, interleukin‐6; lnc‐KCNQ1OT1, long non‐coding RNA potassium voltage‐gated channel subfamily Q member 1 (KCNQ1) opposite strand 1

Lnc‐KCNQ1OT1 was negatively correlated with CRP (*r* = −0.386, *p* < 0.001), TNF‐α (*r* = −0.332, *p* < 0.001), IL‐1β (*r* = −0.319, *p* < 0.001), and IL‐6 (*r* = −0.255, *p* = 0.006) levels (Figure 3A–D).

Moreover, decreased lnc‐KCNQ1OT1 expression was correlated with the occurrence of diabetes (*p* = 0.019) (Figure S1D). However, no correlation was found between lnc‐KCNQ1OT1 expression and other clinical features (all *p* > 0.05) (Figure 1A–C,E–G).

### 3.5 Discrimination of sepsis deaths by lnc‐KCNQ1OT1, inflammatory indices, and SOFA and APACHE II scores

**FIGURE 4:**
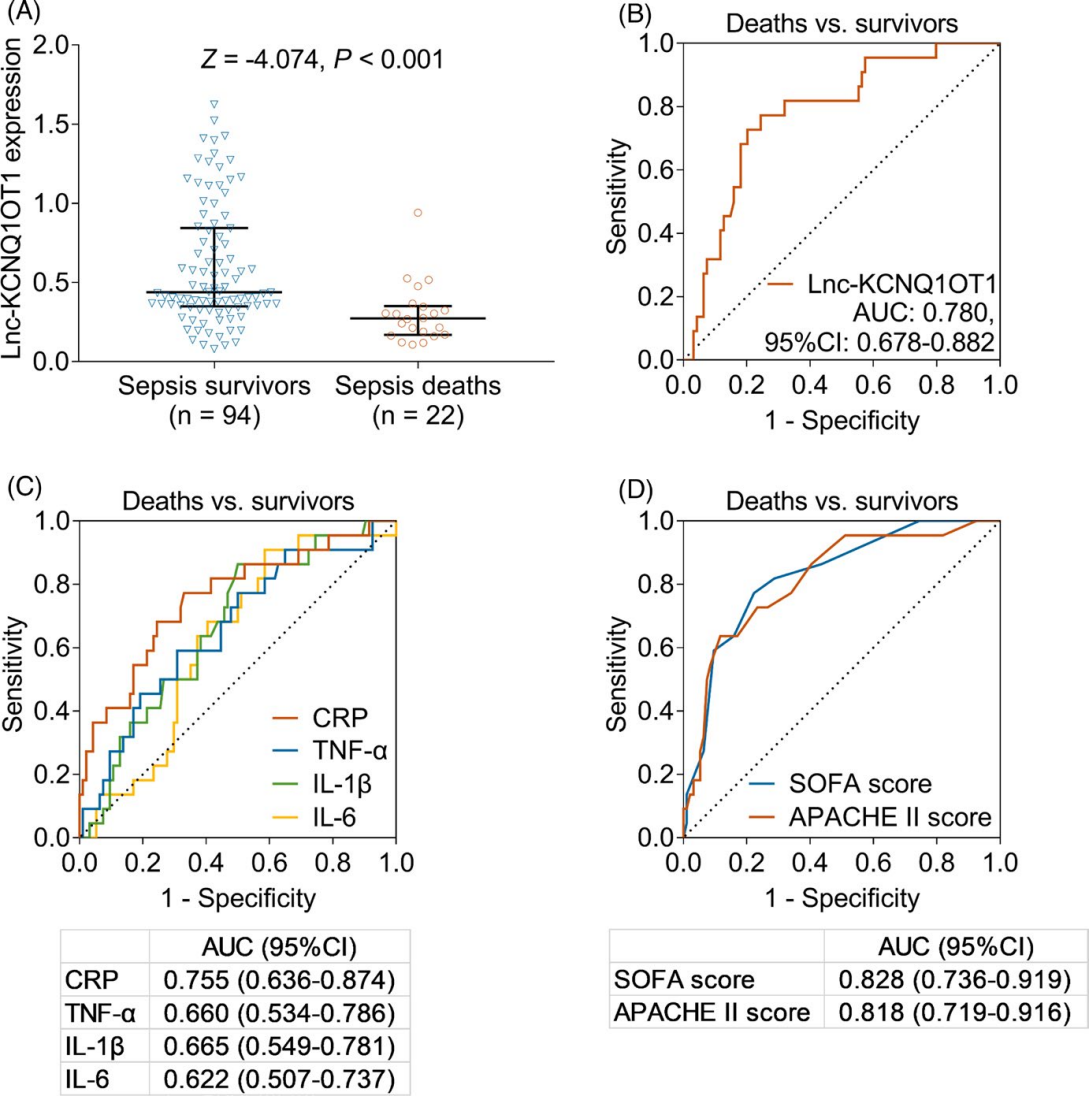
Ability of lnc‐KCNQ1OT1, inflammatory indices, and SOFA and APACHE II scores in predicting mortality risk in sepsis patients. Comparison of lnc‐KCNQ1OT1 levels between sepsis survivors and sepsis deaths (A); discriminatory ability of lnc‐KCNQ1OT1 (B), CRP, TNF‐α, IL‐1β and IL‐6 (C), as well as SOFA score and APACHEII score (D) to distinguish sepsis deaths from sepsis survivors. CRP, C‐reactive protein; TNF‐α, tumor necrosis factor alpha; IL‐1β, interleukin‐1β; IL‐6, interleukin‐6; lnc‐KCNQ1OT1, long non‐coding RNA potassium voltage‐gated channel subfamily Q member 1 (KCNQ1) opposite strand 1; SOFA, sequential organ failure assessment; APACHE II, acute physiology and chronic health evaluation II

Multivariate Cox regression analysis showed that higher lnc‐KCNQ1OT1 levels were independently correlated with septic death (*p* = 0.009, hazard ratio = 0.017) (Table S1). Furthermore, lnc‐KCNQ1OT1 abundance was lower in sepsis deaths (median [IQR]: 0.272 [0.169–0.352]) compared to sepsis survivors (median [IQR]: 0.439 [0.349–0.844]) (*p* < 0.001) (Figure 4A). In addition, the ROC curve showed that lnc‐KCNQ1OT1 expression had a certain ability to discriminate sepsis deaths from sepsis survivors, with an AUC (95% confidence interval [CI]) of 0.780 (0.678–0.882). In addition, lnc‐KCNQ1OT1 expression was 0.349 at the best cut‐off point, with a sensitivity of 0.755 and specificity of 0.773 (Figure 4B). Moreover, the ROC curve illustrated that CRP (AUC [95%CI]: 0.755 [0.636–0.874]), TNF‐α (AUC [95% CI]: 0.660 [0.534–0.786]), IL‐1β (AUC [95% CI]: 0.665 [0.549–0.781]), and IL‐6 (AUC [95% CI]: 0.622 [0.507–0.737]) all had potential in discriminating sepsis deaths from sepsis survivors (Figure 4C). Additionally, the ROC curve showed that SOFA and APACHE II scores had good ability to differentiate sepsis deaths from sepsis survivors with AUC (95% CI) of 0.828 (0.736–0.919) and 0.818 (0.719–0.916), respectively (Figure 4D).

## 4 DISCUSSION

Several studies have shown that lnc‐KCNQ1OT1 is able to inhibit inflammation and multiple organ dysfunction,^6^, ^8^, ^14^, ^15^ whereas hyperinflammation and multiple organ dysfunction are hallmarks of sepsis.^16^ Thus, we speculated that lnc‐KCNQ1OT1 levels might be dysregulated in sepsis, although related information is scarce. Hence, we compared lnc‐KCNQ1OT1 expression between patients with sepsis and HCs. Surprisingly, we found that lnc‐KCNQ1OT1 abundance was lower in sepsis patients than in HCs. A potential explanation might be that reduced levels of lnc‐KCNQ1OT1 could exacerbate inflammation by regulating the XIAP axis and promoting multiple organ injury by targeting miRNAs (such as miR‐192‐5p and miR‐146a), while inflammation and multiple organ dysfunction often occur in sepsis.^7^, ^14^, ^15^ Thus, lnc‐KCNQ1OT1 expression was decreased in patients with sepsis.

It has been shown that lnc‐KCNQ1OT1 can alleviate multiple organ dysfunction.^7^, ^10^, ^17^ For instance, lnc‐KCNQ1OT1 relieves sepsis‐induced myocardial injury by regulating cardiomyocyte proliferation and apoptosis by modulating the miR‐192‐5p/XIAP axis.^7^ Furthermore, lnc‐KCNQ1OT1 has also been reported to have the capacity to alleviate liver injury.^10^ In addition, lnc‐KCNQ1OT1 ameliorates nerve injury by modulating NLRP3 expression via miR‐30e‐3p.^17^ Considering that sepsis is often correlated with multiple organ dysfunction,^16^ we hypothesized that lnc‐KCNQ1OT1 might be correlated with multiple organ dysfunction in sepsis. Thus, we assessed SOFA scores and subscales in sepsis patients, which revealed that lnc‐KCNQ1OT1 was negatively correlated with SOFA scores and its partial subscales (respiratory system, coagulation, liver, and renal systems) in sepsis patients. One possible explanation might be that lnc‐KCNQ1OT1 can inhibit multiple organ dysfunction (including respiratory function, liver injury, and renal function) through several approaches, such as regulating miR‐381‐3p, miR‐122‐5p/CES2 axis, miR‐506‐3p, and miR‐146.^8^, ^9^, ^10^, ^14^, ^15^ Therefore, a negative association was found between lnc‐KCNQ1OT1 levels and SOFA scores and its partial subscales, suggesting that lnc‐KCNQ1OT1 was negatively correlated to multiple organ dysfunction in sepsis.

Previous studies have illustrated that lnc‐KCNQ1OT1 can modulate inflammation.^6^, ^8^ For example, lnc‐KCNQ1OT1 negatively regulates inflammatory factors (including TNF‐α, IL‐1β, and IL‐6) in sepsis‐induced myocardial injury and intimal hyperplasia.^6^, ^7^ Furthermore, other research has also shown that lnc‐KCNQ1OT1 is able to negatively regulate IL‐6, TNF‐α, and IL‐10 expression in acute respiratory distress syndrome.^8^ However, no relevant research has focused on possible correlations between lnc‐KCNQ1OT1 and inflammation in sepsis. Thus, we explored this issue and found that lnc‐KCNQ1OT1 was negatively correlated with CRP, TNF‐α, IL‐1β, and IL‐6 abundance in sepsis patients, which could be explained by the following: (1) lnc‐KCNQ1OT1 could inhibit the proliferation and migration of vascular smooth muscle cells by overexpressing IκBa, which consequently suppresses inflammatory factors (such as IL‐1β, IL‐6, and TNF‐α) and further decreases inflammation in sepsis^6^; (2) lnc‐KCNQ1OT1 might bind to miR‐381‐3p to regulate E26 transformation‐specific proto‐oncogene 2 expression, which sequentially regulates inflammation in sepsis.^8^ Taken together, lnc‐KCNQ1OT1 levels were negatively correlated with inflammation in sepsis.

Currently, the APACHE II and SOFA scoring systems are the two main prognostic assessments for sepsis patients, while their evaluation indices are relatively complicated.^18^, ^19^ Thus, to explore a more convenient method to predict outcomes in sepsis patients, we evaluated lnc‐KCNQ1OT1 expression in sepsis survivors and sepsis non‐survivors. Surprisingly, we discovered that lnc‐KCNQ1OT1 was decreased in sepsis deaths compared to sepsis survivors, and that its expression could discriminate sepsis deaths from sepsis survivors. Furthermore, ROC curves showed that the capability of lnc‐KCNQ1OT1 in discriminating sepsis deaths from sepsis survivors was better than that of CRP, TNF‐α, IL‐1β, and IL‐6, but relatively weaker than the APACHE II and SOFA scores, indicating that lnc‐KCNQ1OT1 could conveniently discriminate sepsis patients with high mortality risk to some extent. Furthermore, our results also demonstrate feasibility in clinical settings. SOFA and APACHE II scores, combined with quantification of lnc‐KCNQ1OT1 expression, could better predict patient prognosis in sepsis cases.

The present study had several limitations: (i) the sample size was relatively small and might lead to diminished statistical power in analyses; (ii) more comprehensive and in‐depth understanding of mechanisms of lnc‐KCNQ1OT1 involvement in sepsis need to be investigated in the future, which might facilitate the development of lnc‐KCNQ1OT1‐based treatments; (iii) the mean age of enrolled sepsis patients was 58.4 ± 13.4 years, thus these findings might not be applicable in younger sepsis patients; (iv) although the present study was multi‐center, selection bias might still exist; (v) sepsis patients were different from HCs regarding circulating indices, hence disease control in sepsis patients could be completed in future studies; (vi) lnc‐KCNQ1OT1 was lowest in respiratory infection‐induced sepsis in the current study, which should be further analyzed for confirmation; (vii) lnc‐KCNQ1OT1 in plasma, derived from PBMCs, could be explored in sepsis patients.

In conclusion, lnc‐KCNQ1OT1 serves as a potential biomarker for monitoring disease severity and prognosis in sepsis patients, which might consequently improve the management of this disease.

## CONFLICTS OF INTEREST

The authors declare no potential conflicts of interest with respect to the research, authorship, and/or publication of this article.

## Supporting information

Fig S1

Table S1

## Data Availability

The datasets generated during and/or analyzed during the current study are available from the corresponding author on reasonable request.
