# Supplementary figures and images for: Potential of long non‐coding RNA KCNQ1OT1 as a biomarker reflecting systemic inflammation, multiple organ dysfunction, and mortality risk in sepsis patients
Source: J Clin Lab Anal. 2021 Nov 10;35(12):e24047. doi: 10.1002/jcla.24047 (PMC8649371; doi:10.1002/jcla.24047)

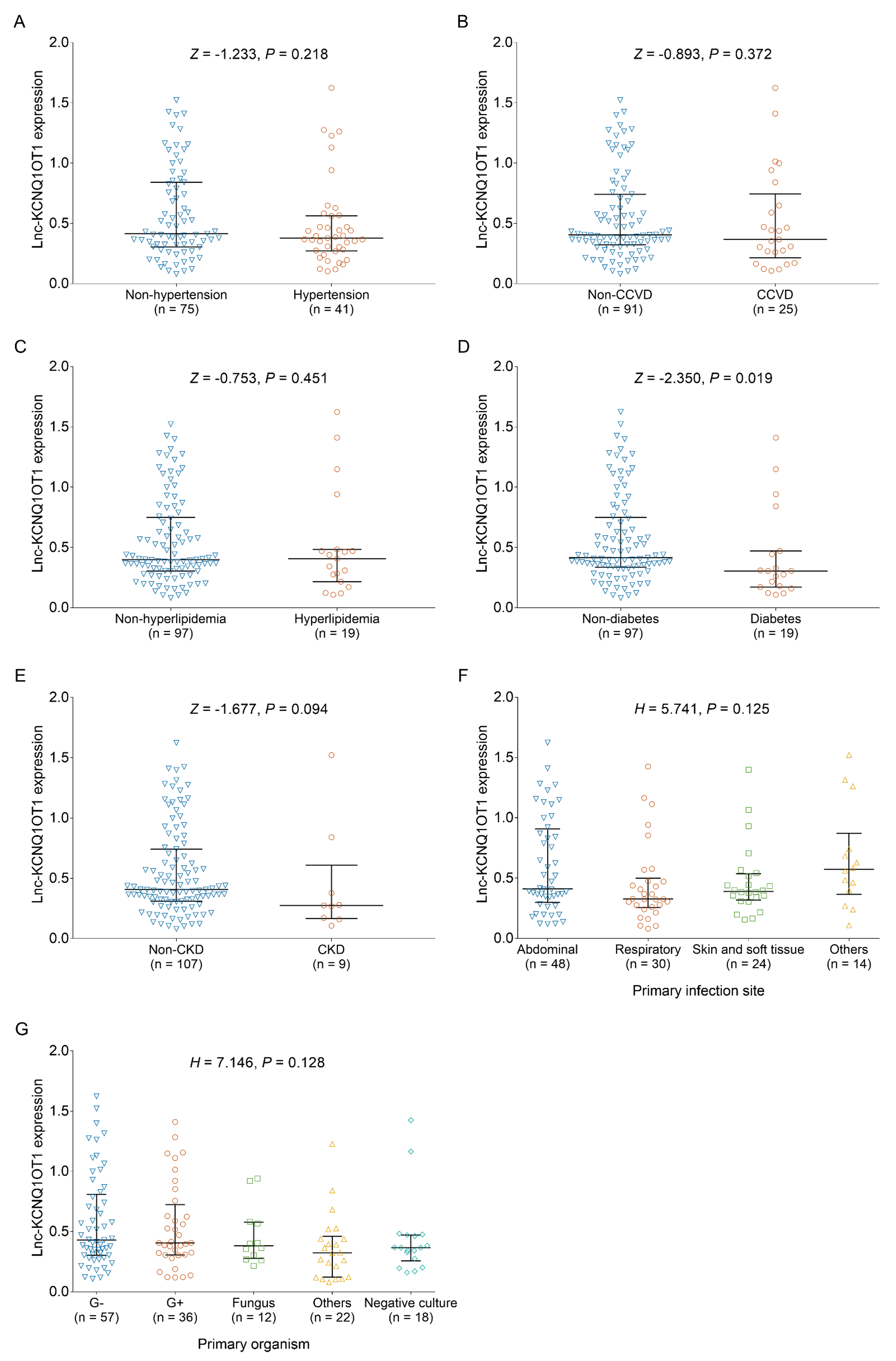

Supplement: Supplementary file 1 — Fig S1 [file JCLA-35-e24047-s001.tif]
