# Supplementary material for: Potential of long non‐coding RNA KCNQ1OT1 as a biomarker reflecting systemic inflammation, multiple organ dysfunction, and mortality risk in sepsis patients
Source: J Clin Lab Anal. 2021 Nov 10;35(12):e24047. doi: 10.1002/jcla.24047 (PMC8649371; doi:10.1002/jcla.24047)
Supplement: Supplementary file 2 — Table S1 [file JCLA-35-e24047-s002.docx]

**Supplementary table 1.** Factors affecting 28-day septic mortality by Cox's proportional hazards regression analysis

| Items | *P* value | HR | 95%CI | |
| --- | --- | --- | --- | --- |
|  |  |  | Lower | Upper |
| **Multivariate Cox's regression analysis** |  |  |  |  |
| Higher lnc-KCNQ1OT1 | 0.009 | 0.017 | 0.001 | 0.358 |
| Higher age | 0.005 | 1.065 | 1.019 | 1.114 |
| CKD (yes vs. no) | 0.008 | 6.403 | 1.633 | 25.104 |
| Higher APACHE II score | <0.001 | 1.270 | 1.151 | 1.401 |
| Primary organism G+ (yes vs. no) | <0.001 | 9.753 | 2.975 | 31.975 |
| Primary organism Fungus (yes vs. no) | 0.001 | 15.499 | 3.022 | 79.493 |

HR, hazard ratio; CI, confidence interval; lnc-KCNQ1OT1, long non-coding RNA potassium voltage-gated channel subfamily Q member 1 (KCNQ1) opposite strand 1; CKD, chronic kidney disease; APACHE II, Acute Physiology and Chronic Health Evaluation II.
